# A Right‐Wing Populist Turn in the Conservative Party of Canada? Continuities and Ruptures Under the Leadership of Pierre Poilievre (2022–2025)

**DOI:** 10.1111/cars.70021

**Published:** 2025-11-23

**Authors:** Efe Peker, Emily Laxer, Rémi Vivès

**Affiliations:** ^1^ School of Sociological and Anthropological Studies, School of Political Studies University of Ottawa Ottawa Canada; ^2^ Department of Global and Social Studies York University, Glendon Campus Toronto Canada; ^3^ Department of Economics, Business and Mathematics York University, Glendon Campus Toronto Canada

**Keywords:** Canadian politics, Conservative Party of Canada, Donald Trump, Pierre Poilievre, political communication, right‐wing populism

## Abstract

Since his election as leader of the Conservative Party of Canada (CPC) in 2022, Pierre Poilievre has been associated with populism in media and political discourse, with implicit and explicit comparisons to Donald Trump. This article investigates the validity of such assessments by applying “complex” theories of populism, which conceptualize populism as an interplay of ideology, communication style, and organizational strategy. Drawing on a qualitative content analysis from the 2022–2025 period, we examine the extent to which Poilievre adopts populist repertoires or reflects continuity with Canadian conservatism. The findings suggest an uneven picture: while his core ideological and policy positions remain largely rooted in Harper‐era neoliberalism and law‐and‐order priorities, Poilievre's communication style—marked by anti‐elite rhetoric, heightened depictions of societal crisis, and combative messaging—reflects a discernible populist turn. Organizationally, Poilievre maintains CPC structures but seeks to reshape them through stricter party discipline, outsider narratives, and unmediated digital outreach strategies that antagonize traditional media. Beyond the Canadian case, the article contributes to theoretical debates by operationalizing a multidimensional framework for the analysis of contemporary populist politics.

## 1 Introduction

On February 5, 2022, as the blockades of the Freedom Convoy were entering their third week, Conservative MP Pierre Poilievre, an ardent supporter of the protests, published a video on social media launching his campaign to lead the Conservative Party of Canada (CPC): “I'm running for Prime Minister to give you back control of your life. Sign up now to help me replace Trudeau & restore freedom” (Poilievre 2022). Seven months later, Poilievre was elected CPC leader with close to 70 percent of the party members’ votes, the largest share ever in a CPC leadership election (Radio‐Canada 2022). By late 2024, Poilievre had earned the CPC a whopping 25‐point lead over Prime Minister Justin Trudeau's Liberal Party of Canada (LPC) (IPSOS 2024). Yet at the turn of 2025, following Trudeau's resignation as party leader and Donald Trump's return to power with tariffs and annexation threats targeting Canada, the Conservatives’ polling lead vanished. In the federal election held on April 28, 2025, the Liberals led by Mark Carney won a minority government and the popular vote—a first for the party since 2015. Although the Conservatives significantly increased their vote share and seats, it was not enough to form government. Poilievre was defeated in his own riding.

Throughout this meteoric rise to political prominence followed by a startling electoral defeat, the labels “populist” and “populism” have been widely used in journalistic and political spheres, mostly in pejorative terms, to describe Poilievre's politics. Much of this characterization referred to his anti‐elite rhetoric targeting the “gatekeepers” of the political establishment—in the Liberal government, the bureaucracy, the media, lobbyists, the Central Bank of Canada, among many others—whose self‐serving “woke” agenda, as he put it, deprives Canadians of the prosperity, liberty, and safety they deserve (Graves and Maher 2022; Lagacé 2022). Comparisons with Donald Trump were drawn, especially on Poilievre's flirtation with the far right and conspiracy theories, combative communication style that stokes divisions between “us” and “them”, and tendency to reduce complex political issues to slogans and soundbites (Hachey 2022; Sirois 2025; Wherry 2022). In response, most conversative critics denied such attributions of populism and comparisons to Trump as “an elitist scare tactic” (Maddeaux 2022) or as a sign of “Liberal desperation” (Taube 2023). Other conservatives held that populism is inevitable in a political reality where the “elites abandoned the working class” (Kheiriddin 2023). These debates reflect a lack of agreement and specificity as to the meaning of populism and whether it is a positive or negative force for democracy.

This article brings clarity to these discussions by applying prevailing social scientific conceptions of populism to the case of Poilievre and contextualizing his politics within the tradition of Canadian conservatism to highlight continuities and ruptures. To realize this objective, we adopt a “complex” approach to populism, which distinguishes between its multiple dimensions and investigates the relationships among them (Diehl 2022). We apply this approach to a qualitative analysis of Poilievre's discourses, texts, and videos found within social media platforms, parliamentary records, public speeches, campaign materials, and news media coverage. Our time frame is January 2022 to April 2025, which takes us from Poilievre's bid for the CPC leadership to his party's electoral defeat. Throughout, we refrain from conceptualizing populism as a *category of practice*—that is, the way the term is used and understood by political actors and commentators, often in normative tones to discredit opponents—and instead employ populism as a *category of analysis*, namely, a conceptual tool for investigating its multidimensional nature in a Canadian case study (Brubaker and Cooper 2000).

We begin with an overview of the theoretical literature on populism. Next, we outline the evolution of federal conservatism in Canada since the 2000s, focusing on the Harper years (2006–2015), during which Poilievre undertook important roles in the CPC. Following a note on methodology, we present our empirical findings. Although we primarily examine Poilievre as part of the trajectory of Canadian populism and conservatism, we draw occasional comparisons with Donald Trump, where relevant, to provide contextual clarity. Finally, we discuss implications for the political sociology of Canada as well as for the broader theoretical literature on populism.

## 2 “Complex” Theories of Populism

Social scientists agree that populism entails division and conflict between the “people” and the “elite”. From this point on, disagreements ensue on at least three issues (Moffitt 2020). The first is whether populism is primarily an ideational, communicational, or organizational phenomenon^1^. The second is whether populism is binary or gradational: can we neatly distinguish populists from non‐populists, or can populism be practiced by political actors in varying degrees? The third is whether populism is a democratizing force, or a pathway to illiberal rule.

Regarding the first question, the ideational approach defines populism as “a thin‐centered ideology that views society as separated into two homogeneous and antagonistic camps, ‘the pure people’ versus ‘the corrupt elite,’ and which argues that politics should be an expression of the *volonté générale* (general will) of the people” (Mudde and Rovira Kaltwasser 2017, 6). As a “thin” ideology, populism needs to borrow from “thick” ideologies (such as conservatism, socialism, etc.) to develop its full expression, which might combine right‐ and left‐leaning elements. In doing so, it amplifies grievances against variously defined elites, simplifies politics into a moral binary, and flexibly adopts content from “host” ideologies. This means, as the empirical section will further clarify, that an ideational analysis has to take into account not only the ‘thin’ but also the ‘thick’ components of a given leader's worldview as distinct yet intricately related categories (Neuner and Wratil 2022).

Claiming that populism lacks the structural features of even a thin ideology, notably a coherent belief system or political program (Aslanidis 2016), the second approach instead defines it as a communicational style. This perspective focuses on how political actors utilize discursive and performative tools to make claims on behalf of the “people” against the “elites”. These tools may include their “folksy” speech, attire, and cultural codes, transgressive behavior and language to challenge political norms and opponents, and highly emotional/dramatic performances to perpetuate a sense of crisis (Ostiguy et al. 2021). In this regard, while conceptualizing populism as “a set of rhetorical operations” rather than an ideology, the stylistic approach likewise acknowledges that populism attaches itself to diverse ideological traditions, “taking on the political coloration of its surroundings” rather than functioning as a self‐contained system (Taguieff 1997, 4).

Finally, the organizational approach views populism as a strategy, employed by a charismatic political figure, to gain and maintain power. This strategy revolves around a personalistic leader who seeks to bypass traditional institutional structures and checks and balances (parties, bureaucracy, media, courts, etc.) to mobilize mass constituencies through unmediated links. Claiming to directly represent the “people”, the populist leader often transcends party organization to incite permanent collective action against variously defined “elites” (Weyland 2017).

Recognizing that a leader or a party can adopt populism unevenly in their ideologies, communication styles, and organizational strategies, an emerging “complex” theory of populism, which we adopt in this article, proposes to study the three components *together* for the purpose of empirically demonstrating their nature and relationships (Diehl 2022; Diehl and Bargetz 2024). In so doing, this approach also answers the second question raised above on whether populism is binary or gradational. Instead of referring to populists versus non‐populists (for instance, Pappas 2019a), the “complex” outlook offers an “understanding of how populism is distributed across the political spectrum” by examining leaders’ and parties’ mobilization of populist repertoires—ideas, styles, and strategies—“in different manners and degrees” (Diehl 2022, 509, 518).

As for the third question, namely, populism's relationship to democracy, scholars disagree as to whether populism is by definition an anti‐pluralist, majoritarian, and illiberal threat to democratic practice and institutions (Müller 2016) or an opportunity to reinvigorate democracy through the inclusion of marginalized groups and increasing government accountability (Tarragoni 2019). This debate is often linked to the distinction between left‐ or right‐wing populisms (Huber and Schimpf 2017), and the extent to which a populist movement frames the “people” as an ethnocultural group (*ethnos*), a socioeconomic underdog (*plebs*), or a politically sovereign community (*demos*) (Brubaker 2017). Rather than taking an a priori position, we recognize in this article that the negative and positive consequences of populism would depend on context and on the specific ways and degrees in which its ideas, styles, and strategies are deployed by a leader or movement (Rovira Kaltwasser 2012).

## 3 Canadian Conservatism in the New Century

Contemporary conservatism in Canada has been characterized as bringing together three principal socio‐ideological currents: neoliberalism, social traditionalism, and populism (Farney and Rayside 2013; Lewis and Everitt 2017). The first prioritizes small government, lower taxes, deregulation, and privatization. The second embraces conventional gender and family norms, religion, social order, and cultural community. The third holds that ordinary people and their “common sense” should directly guide policymaking, as political institutions, parties, experts, the media, and “special interest groups” are inherently suspicious.

The federal election of 1993, which brought a dramatic end to the almost decade‐long Progressive Conservative^2^ (PC) government led by Brian Mulroney, was a major turning point for the reconstruction of conservatism in Canada. The challenge from the right came from Preston Manning's Alberta‐based Reform Party that won 52 seats, against two for the PC. Although largely aligned with the principles of Mulroney's neoliberal agenda, including free trade and cuts to social programs, Reform criticized the PC for failing to curb debt and deficits, for introducing the deeply unpopular Goods and Services Tax (GST), and for privileging central Canadian interests over the concerns of Western taxpayers. Most of Reform's constituents viewed the PC's positions on abortion, same‐sex marriage, and capital punishment as too permissive; policies on bilingualism, immigration, and multiculturalism as too Liberal‐like; and appeals to Québec in the Meech Lake and Charlottetown Accords as too conciliatory (Harrison and Krahn 1995). Resting on a long‐standing tradition of Western alienation (McLean and Laxer 2023; McLean et al. 2024), Reform's populist regionalism helped frame these resentments to target federal parties and bureaucrats as “tax grabbing elites” that “favoured immigrants, native peoples, francophone Québécois, and special privilege‐seeking women's groups and gays over ‘ordinary working people’ in the distribution of state resources” (Laycock 2012, 50). Manning himself defined Reform as a “populist political party” to mobilize “the common sense of the common people” against the federal power bloc by promoting direct‐democratic, “bottom‐up decision‐making processes” rather than traditional party organization (Flanagan 2009, 8, 22).

Although Reform increased its number of seats to 60 in the 1997 federal election to become the official opposition, it failed to garner support outside Western provinces. Subsequent efforts to expand its geographic reach, including rebranding the party as Canadian Alliance in January 2000, did little to reverse the trend, as confirmed by a poor showing in the November elections that year. In March 2002, Stephen Harper was elected to lead the party, and soon after, Alliance and the PC merged to form the CPC with Harper at the helm. Harper had been a founding member of Reform and Manning's chief policy officer, winning his first seat in 1993. He did not seek re‐election in 1997, mainly because he believed Manning's populism was not based on a principled conservative vision (Boessenkool and Speer 2015). Presiding over the conservative lobby group National Citizens Coalition between 1998 and 2002, Harper maintained his close affiliation with the Albertan right, advocating for a “stronger and much more autonomous Alberta”, a province that he praised as shaped by “American enterprise and individualism”, unlike the Canada of the Liberals that he described as “a second‐tier socialistic country” (Harper 2000).

Scholars have argued that as Prime Minister (2006–2015), Harper exhibited some populist tendencies, which they variously qualified as “market populism” (Sawer and Laycock 2009), “mainstream populism” (Snow and Moffitt 2012), “neoliberal populism” (Budd 2021), “penal populism” (Kelly and Puddister 2017), and “authoritarian populism” (Carlaw 2017). Harper himself embraced the concept of populism, yet as a positive effort to place “the wider interests of the common people ahead of the special interests of the privileged few”, and has repeated his calls for “populist conservatism” as the path to follow for the CPC (Harper 2018, 13, 77). Critical works, however, focus on the Harper CPC's assertive economic rhetoric, which condemned the welfare state as an elite project pampering “special interest groups” to help justify the party's neoliberal cuts and deregulation efforts (Budd 2021; Sawer and Laycock 2009). Likewise, Harper's policy discourses on issues as diverse as crime, childcare reform, the long‐form census, and the arts were, according to analysts, anchored in antagonism to experts, courts, and other institutions portrayed as undermining the “common sense” of ordinary people (Kelly and Puddister 2017; Snow and Moffitt 2012). Others observe that although the CPC mostly shed the anti‐diversity leanings present in Reform and implemented an “ethnic outreach” strategy toward minority communities (Taylor 2021), the party incrementally undermined immigration and multiculturalism policies in the background, and promoted majority cultural tropes to satisfy its base. These included reducing family reunification programs, taking strong positions on refugees and undocumented migrants, and placing greater nationalistic emphasis on the military, the monarchy, and shared history and values rooted in British heritage (Abu‐Laban 2014; Carlaw 2017; Forcier and Dufour 2016; Kwak 2019).

Pierre Poilievre was a first‐hand witness and participant during the reconfiguration of Canadian conservatism in the 1990s. Born and raised in Alberta, he joined Reform at a young age and became a party delegate in the 1996 national convention, when he was only 17. As a student at the University of Calgary, he presided the Young Tories club, and in line with the Reform position, was involved in multiple disputes with the youth members of the PC, such as future leadership competitor Patrick Brown (Dryden 2022). He moved to Ottawa in the early 2000s to become a political advisor in the Canadian Alliance, and in 2004, at the age of 25, was elected as an MP in Ottawa from the Harper‐led CPC. In his more than two‐decade career as an MP, Poilievre gained national attention for his partisan attacks on people and policies he opposed, leading critics to dub him the Conservatives’ “attack dog” (Clark 2022; Proudfoot 2022). He served as Harper's Minister for Democratic Reform (2013‐15) and Minister of Employment and Social Development (2015), and following Harper's defeat in 2015, as the CPC's Shadow Minister for Finance (2017‐22). During COVID‐19, Poilievre stood out as a fervent critic of Trudeau's pandemic‐related policies, which he qualified as “unfair, unscientific bullying” (Graves and Maher 2022). At the height of the Freedom Convoy in early 2022, he was convinced that the time was right to run for the leadership of his party. In the empirical section below, we take a close look at the period that followed, focusing on the extent to which the ideas, style, and strategies adopted by Poilievre maintained or departed from earlier iterations of Canadian conservatism with regard to populist repertoires.

## 4 Analyzing Poilievre's Ideas, Style, and Strategies

### 4.1 Note on Methodology

Empirically distinguishing between the ideational, stylistic, and strategic aspects of populism is notoriously difficult, as the beliefs, rhetoric, and actions of political figures overlap, fuse, and reinforce each other. The subsections below will attest to this difficulty. The three components nevertheless remain useful analytical and organizational tools for a theory‐informed case study (Yin 2018), which qualitatively analyzes the interplay between Poilievre's ideological tendencies, discursive practices, and strategic actions in the 2022–2025 period. Our method involves the close reading of a broad array of primary sources—including Poilievre's speeches, social media content, campaign materials, and parliamentary interventions—and secondary sources such as news, media commentary, and political biographies in order to identify salient themes pertaining to populism. This approach follows a tradition of qualitative case study methods, wherein a rich selection of materials about the object of study is analyzed in context to illuminate meaning‐making routines, discursive constructions, and the choices of the key actors in question (George and Bennett 2005). Our goal is not to quantify instances of populist practice, nor to present a comprehensive chronology of the 3‐year period, but rather to thematically assess how the three dimensions of populism manifest and interact in a Canadian case. This strategy allows for theoretical engagement while remaining grounded in empirical observation to offer insights for and beyond the case at hand.

### 4.2 Ideology

Informed by the notion that researchers should “properly distinguish populism from ‘what it travels with’” and “dedicate attention to the connection between populism and its host ideologies” (Hunger and Paxton 2022, 617, 630), this subsection focuses more on Poilievre's “thick” ideological commitments and policy positions than on the “thin” anti‐elitism associated with populism. (The subsequent subsection sheds more light on Poilievre's anti‐elitism, which can operate simultaneously as an expression of a thin‐centered worldview *and* as a discursive tool^3^). We find that when it comes to substantive, “thick” ideologies and policies, radical right themes that characterize Trump's leadership—economic protectionism, nativist immigration restrictions, unilateralism that challenges global institutions, sweeping anti‐abortion and anti‐LGBTQ measures, and authoritarian executive power (Graham 2025; Mudde 2022)—do not prominently feature in Poilievre's political agenda. Instead, Poilievre seems to align more closely with Harper‐era conservatism, albeit with more pronounced libertarian undertones, than with Trumpism^4^.

Like Harper, Poilievre hails from the intellectual tradition of the “Calgary School”, with primary emphasis on economic policy defined by free markets, small government, and low taxes, firmly grounded in a Western regionalism skeptical of federal centralization and welfare provision^5^. A long‐time admirer of Milton Friedman and Friedrich Hayek, and “already associated with a more libertarian current of Canadian conservatism” since his early political career (Montigny 2025, 65), Poilievre has consistently placed fiscal and monetary discipline at the heart of his rhetoric. His tweets maintain a sharp focus on the economy, addressing the “people” primarily in economic, rather than cultural or political terms (Laxer et al. 2025, 12‐13). He repeats grievances about skyrocketing inflation, interest rates, housing costs, taxes (especially on carbon), and bureaucratic red tape, for all of which Trudeau/Carney Liberals and affiliated elites are directly blamed. Poilievre's systematic framing of the “people” as socioeconomic underdogs pitted against the establishment, complemented by his libertarian, anti‐institutional gestures like his pledge to fire the Bank of Canada governor and endorsement of cryptocurrencies, mark a shift from the Harper years. Despite such novelties, explored further in the next subsection, Poilievre's economic vision since he assumed the CPC leadership has been generally applauded in conservative circles as a conventional pro‐market agenda that promises to deregulate, among other things, the oil and gas sector (National Post View 2023).

On immigration and multiculturalism too, Poilievre resembles Harper. Although 58 percent of Canadians (and 80 percent of Conservatives) believe that “overall there is too much immigration in Canada” (Environics 2024), and three‐fourths of the population (and 84 percent of Conservatives) believe that “immigration is contributing to the housing crisis” (Léger 2023), Poilievre has avoided explicitly problematizing immigration or immigrants. Instead, the 2025 party platform (CPC 2025b, 17) promised to “restore order to immigration”, “*similar to the levels under the Harper government*” (our emphasis), by keeping “the rate of population growth below the rate of housing growth, job growth, and health care accessibility” to ensure fairness “for Canadians and newcomers alike”. This is a far cry from Trump's incendiary rhetoric. Instead, Poilievre's ethnic outreach in metropolitan suburbs highlights “work, family, freedom, tradition” as values shared both by Conservatives and immigrants, and his Venezuelan‐born Quebecer wife Anaida Poilievre plays an active part in boosting his image as a pluralist politician (Taylor 2023). Also, regarding diversity, Poilievre maintains a pro‐choice and (Tasker 2023) pro‐gay rights stance (although he voted differently as a younger MP), and apart from his support for banning puberty blockers for minors and remarks on “parental rights” against “gender ideology” in schools, he seeks to avoid gender identity‐related matters. This is in marked contrast with his party's delegates who overwhelmingly voted to make this a key policy issue (Vastel 2024).

Rather than methodically targeting immigrants or ethnic/sexual groups *à la* Trump, Poilievre attacks the elite establishment's “wokeness”, understood as the opportunistic and authoritarian exploitation of minority identities to consolidate power. Poilievre defines wokeness as a divide‐and‐conquer ideology that has “only one purpose: control. It is designed to divide people by race, gender, ethnicity, religion, vaccine status and any other way one can divide people into groups” (House of Commons Debates 2023). Imported from the American culture wars of the past decade, the label “woke” is a new addition to the CPC vocabulary, serving ideological as well as rhetorical functions. Ideologically, it aligns Poilievre with “freedom” against Liberals’ perceived government overreach, and allows him to echo, without attacking minorities themselves, Trumpian anti‐diversity themes that appease the hardliner segments of the Canadian right (Leman‐Langlois et al. 2024). Yet parliamentary records show that Poilievre uses the concept much more broadly to critique government policies on the environment, crime, housing, drugs, internet regulation, borders, and gun control, among others. “Woke”, in other words, is not strictly about identity‐related ideologies, and has become a catch‐all rhetorical tool for targeting any liberal/left policy approach with which the Conservatives disagree.

Regarding nationalism and British heritage as well, there are overlaps with the Harper years. In his victory speech as the newly elected leader of the CPC, Poilievre said: “I feel a small catch in my throat when I utter the words that no leader has stated in this country for over seven decades: ‘God save the king!’” (Aiello 2022). A year later, at the Conservative Convention in Québec city, he accused Trudeau of being “ashamed of our common heritage” and wanting to “cancel our proud history”. Poilievre saw the new Canadian passport design unveiled in May 2023 a case in point, because “Justin Trudeau erased it [our heritage] from the passport—he also erased the Royal Twenty‐Second Regiment, Vimy, and the other glorious moments of our history. He says we have no identity as a country” (National Post 2023). In his 2025 Flag Day speech, he vowed to reinstate Sir John A. Macdonald's statues, strengthen penalties for defacing monuments, restore military and historical symbols to the passport, and “stop the war on our history” (CPC 2025a).

Notwithstanding such continuities, which also include a tough‐on‐crime approach and looser gun laws, Poilievre's ideological positions are fluctuating in other policy areas, at times leaning toward Trump‐like anti‐globalization themes. Long before the 2025 US trade war, Poilievre offered protectionist remarks on the economy, including that “we shouldn't fund jobs for their [other countries’] workers”, which could suggest that “he is willing to turn his back on the free‐trade ethos his party once championed to score political points” (Yakabuski 2023). Security is another topic where Poilievre is prone to framing national interest and global institutions in zero‐sum terms: Instead of “another gabfest at the UN or in—God‐forbid—Davos, … we secure Canada … by standing on guard for our country and rebuilding our military here at home” (National Post 2023). As a jab to the Liberals, his 2025 election platform proposed “defunding foreign aid to dictators, terrorists and global bureaucracies” (CPC 2025b, 13). Similar examples include his party's opposition in 2024 to a trade deal with Ukraine (due to mention of “carbon pricing” in the text), and his (not‐so‐) implicit endorsement of conspiracy theories on the “great reset” and the World Economic Forum (WEF, exemplified below). Such attacks hint at a nationalist appeal that contrasts Canadian priorities with foreign influence, while also drawing on a populist framing of amorphous, unaccountable elites. Nevertheless, due to the ambiguity of these positions, “questions abound as to whether Poilievre will embrace the traditional Conservative” path, “or follow a more isolationist approach that mirrors the thinking in some right‐wing circles around the world” (Levitz 2023).

Poilievre invited further comparisons to Trump by intensifying certain policy stances ahead of the 2025 election. In November 2024, after a pro‐Palestinian demonstration in Montréal turned violent, Poilievre blamed Trudeau's “toxic woke identity politics” and added: “You opened the borders to terrorists and lawbreakers and called anyone who questioned it racist” (Poilievre 2024). During his “Canada First—For A Change” electoral campaign, Poilievre pledged not only to deport illegal border crossers, but also to remove individuals on temporary visas who engage in supposedly unlawful activities such as participation in “hate marches”. Extending his borrowing from south of the border, he targeted equity, diversity and inclusion initiatives “to put an end to the imposition of woke ideology in the federal civil service and in the allocation of federal funds for university research” (Thériault 2025). Poilievre has also pledged using the notwithstanding clause to override Charter protections, an extraordinary step never before taken by a federal government, to impose mandatory life sentences for fentanyl traffickers and consecutive sentences for multiple murderers. Such moves have been interpreted by some as indicative of an authoritarian and exclusionary bent in Poilievre's vision (Delacourt 2024). It remains to be seen whether these statements reflect a more coherent, “thick” ideological transformation or rather a strategic, issue‐specific turn shaped by the electoral context.

### 4.3 Communication Style

Closely related to *what* Poilievre says or writes on various platforms is *how* he communicates. According to Conservative political strategist Dan Robertson, Poilievre “just happens to be more aggressive in the way that he communicates but there's nothing out of the ordinary about Poilievre's political views. They're all recognizably conservative within a Canadian context” (Thomson 2023). Although Poilievre's abstruse remarks on the global order and more assertive election rhetoric cited above may suggest a more fluid ideological reality, Robertson is right that Poilievre's real distinguishing feature is a belligerent anti‐elitist style that differs from the Harper era.

Moffitt (2016, 45) holds that at the core of populist communication are “the people’ versus ‘the elite’; ‘bad manners’; and the performance of crisis, breakdown and threat”, often by a charismatic leader^6^. Other stylistic traits include the use of “simple language, directness, and oversimplification of political issues”, “ordinary vocabulary and vulgar expressions”, Manichean framing of “us” versus “them”, “conflicting and polarizing” rhetoric, “highly emotional and dramatic” performances, “offensive and aggressive language to describe … opponents”, and an exclusive claim to represent the people's “common sense” through a “perspective of looking up from below” (Diehl 2024, 28).

Poilievre's discursive style, tightly interwoven throughout his career with his “thin” yet unwavering ideology of anti‐elitism, exhibits these criteria. Soon after his move to Ottawa in the early 2000s, he declared that there were two groups in the Canadian capital: “The one made up of the hoity‐toity chattering class of special interest groups and lobbyists” and the “real Ottawa” made up of “real people” he claimed to represent (Forrest 2022). In 2014, as the Conservative government undertook an election reform, then‐Minister Poilievre attacked Elections Canada for craving “more power, a bigger budget and less accountability”, an institution he deemed as consisting of “Liberal lapdogs” seeking to manipulate the vote (Wherry 2019). In the midst of the pandemic in November 2020, he started a petition to “stop the great reset” supposedly undertaken by Trudeau, because “Canadians must defend themselves against global elites who prey on people's fears and despair in order to impose their power grab” (Proulx 2020). In August 2023, Poilievre similarly iterated that “it is time we rejected the globalist elites of Davos and brought back the common sense of ordinary people” (Djuric 2023). In a campaign speech in April 2025, he told his supporters that while they worry about a tax audit from the Canadian Revenue Agency, “you learn that global liberal elites are getting away without paying anything at all. One of these individuals happens to be … Mark Carney” (Sandor 2025).

Rarely evident in Harper's discourse, this long‐standing populist rhetoric appears to have further intensified as part of Poilievre's bid to attract low‐ to middle‐income voters whose economic precarity deepened in the post‐COVID context. Montigny (2025, xi), for instance, writes that Poilievre is “busy courting the working class … hunting on the left, on the NDP's turf”^7^. Indeed, Poilievre's anti‐elitism fuses ideological and rhetorical elements to embody the ordinary, hardworking “people” against a combination of economic, political, and cultural figures and institutions. Examples include “the media, the pundits, the professors”, the “cozy club of insiders”, “Liberal gatekeepers and corporate oligarchs”, “Laurentian elite liberal media”, “woke liberals”, “lobbyists”, “big bossy Liberal government”, some big‐city mayors, and the “socialist coalition”, among many others. In this framework, experts’ and intellectuals’ views are contrasted with the good judgement of the people: “I think we would all be better off if fewer of these so‐called self‐appointed experts, who consistently get it wrong, were in charge of the country and more of the common sense of the common people were brought forward” (House of Commons Debates 2023). Data from 2022 indicates that in 75.7 percent of all tweets where Poilievre blames an elite, he is referring to the political establishment, even when he is talking about economic or other issues (Laxer et al. 2025).

“Wokes” and “gatekeepers” are powerful stylistic tools to imagine the elites, aligning seamlessly with the libertarian tones of Poilievre's ideology. In Poilievre's communication style, “woke” refers to individuals and groups (“a woke prime minister”, “the woke brigade”, “the woke totalitarian left”, “woke bureaucrats”), parties (“the woke Bloc”, “woke, left NDP‐Liberal mayors”), and institutions (CBC, Canadian Radio‐television and Telecommunications Commission, “woke agencies”, “woke censorship ideology to university campuses”) that force their dogmatic outlook on ordinary people to advance an authoritarian agenda. “Gatekeepers” make up a similarly important aspect of Poilievre's rhetoric. Indeed, the dramatic increase in federal leaders’ use of this term since the Freedom Convoy was driven almost entirely by Poilievre, who used the term between four and seven times per week on X/Twitter during his CPC leadership bid (Vivès et al. 2024). Consistent with his libertarian‐inspired conservatism that endorses small government, Poilievre frames “gatekeepers” as a collective that “prevents the market from developing naturally and organically” to create “additional costs for ordinary Canadians” (House of Commons Debates 2023). This may include “bureaucratic” or “local” gatekeepers and “left NDP‐Liberal mayors” (“the worst gatekeepers of all”), who slow down housing construction through “red tape”. In parliament, Poilievre dismisses different kinds of taxation and/or government regulation as “gatekeepers” obstructing the “natural” flow of things through procedures and deliberation, applied to policy areas as variegated as mining, public health, energy production, immigrant skill certification, Indigenous affairs, online speech, and financial and monetary strategy (e.g., Bank of Canada, Canada Infrastructure Bank). The sometimes‐overlapping categories of “wokes” and “gatekeepers” depict the elites as a coordinated group seeking more power through their cultural and political‐economic influence, respectively.

“Bad manners”, too, have long been part of Poilievre's discursive style. In 2006, he said “f*** you guys” to members of a parliamentary committee; the same year, he made an obscene hand gesture to the elected officials in the House of Commons; in 2008, regarding compensations for residential school survivors, he said “are we really getting value for all of this money?”; in 2009, he compared the carbon tax to a “tar baby”; in 2010, he created a scene on Parliament Hill by blowing an RCMP security check point because he did not want to wait (Vieira and Proulx 2024). As CPC leader in the House of Commons, he exclaimed “W‐T‐F?” (What the f***?) in response to the Liberals’ management of the pandemic application, ArriveCan. He was ejected from parliament for calling Justin Trudeau a “wacko”. His testy exchanges with journalists have gone viral in online right‐wing circles even outside Canada, such as his well‐known undercutting, while eating an apple, of a reporter who asked if he was embracing populism (see RCI 2023), or his recurrent retort to unwelcome inquiries, such as: “You're a tax‐funded mouthpiece to the PMO [Prime Minister's Office] … Our party does not support tax dollars for media outlets, because that's when we end up with biased media like you” (Delacourt 2024).

Another populist element of Poilievre's communication style is his use of hyperboles and superlatives to portray Canada as in crisis or breakdown, at times echoing a Trump‐style “carnage” rhetoric. He insists, for instance, that things have “never been worse” than during the “lost Liberal decade”, that “everything is worse” under Trudeau, and that “everything seems to be broken”. Canada is the “worst economy in the G7” where there is “creeping totalitarianism” and a “war on work”; it has the “worst immigration minister in Canadian history”, and an “incompetent Prime Minister” whom he once referred to as “a Marxist”. In one press conference, he asked, “can you think of anything he hasn't screwed up, like seriously?” (Rana 2024). Finally of note is Poilievre's abundant utilization of catchy slogans. These include personal insults and nicknames such as “Trust‐fund Trudeau”, “Sneaky Carney, he's just like Justin”, “Sellout Singh”, “woke Bloc”, but go beyond: “Fire the gatekeepers”, “Axe the tax”, “Boots not suits”, “#Justinflation”; “ArriveScam”, “Bring it home”, “common sense Conservatism”, “carbon tax cover‐up”, “because you're woke, everyone else is broke”, “take back control of your life”, “vaccine vendetta”, and “Trudeau, he's not worth the cost”, among others. Poilievre's anti‐elite slogans and expressions, which are habitually repeated by other CPC members, often rhyme, and lean on repetitions, alliterations, and wordplays.

### 4.4 Organizational Strategy

In contrast to his communication style, Poilievre's political‐organizational strategies do not overlap, at first glance, with typical cases of populism. A Conservative MP for two decades, Poilievre is far from being a political outsider like Donald Trump in the US or Javier Milei in Argentina, and despite his strong personality and charismatic leadership, he has so far generally upheld rather than undermined the CPC's party structure or procedures. The amorphousness, weakness, and fluidness of political organization associated to populist strategies (Barr 2019), therefore, are not immediately observable in Poilievre's case.

There are, however, signs of change. Politically socialized in the Reform tradition, Poilievre is no stranger to populist modes of organizational strategizing. Reform sought to establish direct, unmediated links with grassroots movements and what Preston Manning called the “common sense of the common people”. An important part of especially early Reform's populism was the skepticism toward turning into a fixed, formal party structure like others, as they aimed “not just at governing within the prevailing system but at changing the system itself” (Flanagan 2009, 22). Although Poilievre is not overhauling the CPC's formal party structure, some of these anti‐institutional, anti‐conformist reflexes shape his political brand. In his 2025 election campaign, for instance, Poilievre made an appeal to direct democracy by proposing a “Taxpayer Protection Act” that would mandate national referendums for any new federal tax increases (CPC 2025b, 5). As Yaroslav Baran (2024), former communications director of the CPC, approvingly writes, “to understand Pierre Poilievre, we need to understand that he cut his teeth as an acolyte of Preston Manning”, especially in his embrace of the “Prairie populist tradition” of “common sense” politics against the “official Ottawa” ran by the “Laurentian consensus” of Ontario and Québec elites. It is through tapping into the uniquely Canadian history of populism that Poilievre fashions himself as an outsider to, and a disruptor of, the Ottawa elites’ schemes, even though he has been an Ottawa MP since his twenties.

Poilievre's attacks on insiders are even occasionally directed at those within his own party. As a young MP, he was a key figure of the “Khmer Bleu” within Harper's CPC, “a semi‐secret group of some of the more right‐wing Conservative MPs” that positioned itself against the party's “Red Tories” and “Quebec caucus”, both seen as Liberal‐like big spenders. The group included many Western MPs as well as figures like Maxime Bernier, and pushed for “a truly conservative agenda” (Lawton 2024, 70). Poilievre's “outsider” image became especially apparent during the Freedom Convoy of 2022, when he unequivocally supported and physically joined the ranks of the truckers in direct opposition to the then‐CPC leader Erin O'Toole. X/Twitter data show that thanks to his direct grassroots appeal, “in terms of both likes and retweets, Poilievre's popularity increased dramatically during the convoy and remained higher in its aftermath”, a performance six times higher than the other candidates in the subsequent CPC leadership race (Vivès et al. 2023, 3‐4).

During that race, Poilievre doubled down on his support for the convoy, accusing Jean Charest, his rival and former leader of the Québec Liberal Party who served as Premier between 2003 and 2012, of being part of the “elites”, and of having “learned about the trucker convoy on the CBC like other Liberals”. He added: “the average trucker has more integrity in his pinky finger than you [Charest] had in your entire scandal‐plagued Liberal cabinet” (CPAC 2022). Another close contender, Patrick Brown, was pushed out of the race by Poilievre's campaign, which paid for the legal fees of a whistleblower who had implicating information on him (Levitz 2023). Conservative MP Alain Reyes, who quit the CPC following Poilievre's election as party leader, claimed he was a victim of “bullying”. CPC members were sent automated text messages to force him to resign, because Reyes had allegedly “decided not to fight Trudeau's inflation with Pierre Poilievre's united team” (Pirro 2022).

In the three years that followed, Poilievre has imposed stringent party discipline within the CPC, with reports of MPs being closely monitored by staffers, discouraged from interacting with other parties or certain media, and expected to strictly adhere to the leader's messaging and slogans (Noel 2024). Such strategies became apparent during the 2025 election, where decision‐making was confined to a small inner circle, and a highly personalized focus was kept on Poilievre at the expense of any other CPC member. This assertive leadership approach leaves little room for autonomy among MPs, yet observers note continuity rather than a break with Harper's style. As a former CPC adviser under Harper observed, “Poilievre went to the Harper school … where message discipline was important” (Noel 2024). Although centralized, controlling, and disciplinarian, Poilievre's approach is not, therefore, conclusive evidence of a distinctly populist organizational turn.

Scholars employing the strategic‐organizational approach note that social media have been crucial in bypassing traditional media to enable “unmediated, quasi‐direct appeals” to the people (Weyland 2017, 58). Poilievre fits this description by favoring social media over what he sees as the liberal favoritism of mainstream outlets. In a message to CPC members soon after assuming the party leadership, he stated: “We can't count on the media to communicate our messages to Canadians. We have to go around them and their biased coverage. We need to do it directly with ads, mail, phone calls and knocking on millions of doors” (Bellavance 2022). It is based on this skepticism that Poilievre very rarely accords radio, television, and newspaper interviews, with the notable exception of media in Québec, where the CPC is drastically underperforming. He has avoided the annual Parliamentary Press Gallery Dinner since becoming CPC leader—a stance mirrored by many of his MPs, after party staffers were seen taking note of which Conservatives attended the event. Poilievre also tends to avoid Parliament Hill reporters, and when he does engage with them, he typically takes only a very limited number of questions, like Harper in his later years (2011–2015).

Unlike Harper, however, who “displayed a certain distance, a reserve” toward the media, Poilievre “throws down the gloves and often responds to reporters as if they were political adversaries” (Bellavance 2022), leading to the tense exchanges exemplified in the previous section. In 2023, Poilievre successfully asked Elon Musk to tag the CBC as “government‐funded media” on X/Twitter, a decision he celebrated with the following words: “Now the people know it's pro‐Trudeau propaganda, not news” (Lagacé 2023) (X has since removed all such tags from media organizations). During the 2025 election campaign, Poilievre imposed strict limitations on media access: journalists were confined to designated areas approximately 25 m from the podium, permitted only a few pre‐approved questions without follow‐ups, and were barred from traveling on the Conservative campaign plane or bus—a departure from longstanding Canadian electoral traditions (Gollom 2025).

Social media thus becomes Poilievre's primary channel of communication, where the non‐traditional medium matches the anti‐institutional message, and the combined slogan of “Axe the Tax, Build the Homes, Fix the Budget, Stop the Crime” is repeated. Especially of note is Poilievre's abundant dissemination of videos on X/Twitter, YouTube, Facebook, and Instagram, which can be grouped in at least four categories. First are videos where Poilievre speaks in public, at the House of Commons and (less commonly) in press conferences or interviews, as well as those featuring speeches and exchanges in community events and rallies—sometimes with emotional background music added. Second are the topical, high production campaign videos that appear like mini documentaries. These generally have a bleak tone and soundtrack highlighting a breakdown or a threat, with Poilievre often doing the voice‐over to talk about problems such as housing, inflation, national debt, agriculture, extorsion, car theft and other crimes, natural resources, and Indigenous relations. Third are videos in which Poilievre speaks directly to the camera, either to deliver a formal message at his desk (related to various holidays, commemorations, etc.), or more candid videos in natural settings (such as a porch, a shed, a car, etc.). In the fourth and final category is a combination of lightly edited news footage and declarations from government officials, intended to demonstrate how bad things are in Canada and how incompetent the current leaders are. These are in addition to videos and ads portraying Poilievre's down‐to‐earth family life with his wife and children, presenting him as relatable and in touch with regular Canadians, a theme that featured prominently in the 2025 election campaign. Although a comprehensive media analysis is beyond the scope of this article, Poilievre's social media use exemplifies an attempt to establish a direct connection with constituencies by removing the intermediaries who cannot be trusted.

## 5 Discussion and Conclusions

Diehl (2024, 24, 25) writes that “the complex concept of populism acknowledges that … populism can manifest itself in communication, ideology, and organization to different degrees”, and it does not necessarily affect these “dimensions … equally or coherently”. Our empirical analysis of Poilievre's politics lends credence to this claim, revealing that the extent to which his approach coincides with populism differs across dimensions.

In terms of “thick” ideological content, Poilievre largely maintains the principal tenets that have defined 21st‐century conservatism in Canada: a neoliberal program seeking to minimize government regulation and taxes (with close ties to the fossil fuel industry), overall positive albeit more reserved (than Liberals) attitude to immigration and multiculturalism, a law‐and‐order agenda with anti‐drug and pro‐gun tones (although he avoided the latter issue during the 2025 election campaign), and a dose of nationalist emphasis on the military and cultural values cherishing the British heritage. On some issues, Poilievre's “thick” ideology is even more progressive: his pro‐abortion, pro‐gay rights stance is manifestly more permissive than what the socially conversative base of the CPC would have preferred. His (rather quiet) opposition to gender‐affirming healthcare and educational practices for minors, and vague defense of related policy changes in some provinces seem to be the only concessions given to that base so far (Tasker 2023).

Yet on other issues, Poilievre does not refrain from flirting with ideological themes further to the right. These include his nods to WEF or pandemic‐related conspiracies, disparaging declarations on global institutions, cryptocurrency endorsement, protectionist takes on the economy, opposition to a trade deal with Ukraine, and use of hidden hashtags such as #mgtow in his videos^8^. He has also drawn comparisons to Trump through proposals to defund universities and federal institutions accused of promoting “wokeness”, expand deportations beyond illegal border crossers, and invoke the notwithstanding clause to impose constitutionally controversial tough‐on‐crime measures.

These ideological oscillations may be a response to electoral imperatives stemming from broader shifts on the Canadian right since the Harper years, including the rise of populist/far‐right/libertarian movements (Johnston 2023; Leman‐Langlois et al. 2024). Founded in 2018, Maxime Bernier's People's Party of Canada, whose federal vote share shrank from 4.9 percent in 2021 to 0.7 percent in 2025, is Poilievre's natural competitor for such constituencies. This often places Poilievre in a difficult balancing act, leading to his occasional nods to more libertarian and/or Trumpian talking points. Yet, for the most part, he has maintained policy positions consistent with the Harper era. In 2024, for instance, Poilievre did not issue a message on the second anniversary of the Freedom Convoy, and on social media, his photo ops with ethnic communities or pro‐Ukraine messages are often met with contempt from groups further to his right. Available evidence suggests that Poilievre does not represent a substantial ideological shift within the CPC toward the radical or far right, or to full‐fledged libertarianism. In other words, the “host” ideology of 21st‐century Canadian conservatism is more intact and recognizable than what can be said of its American counterpart under Trump.

Rather than overhauling the host ideology, Poilievre marries it with his “thin” yet prominent ideology of anti‐elitism, delivered with a suitably combative communication style that marks an unmistakably populist turn. His tireless anti‐elite discourse against “gatekeepers” and “wokes” in the Liberal establishment and its affiliates attests to a striking rhetorical transformation that also accords with his libertarian‐inflected ideological stance that is inherently skeptical of government. Harper was no stranger to taking populist jabs at “experts” and “special interest groups” (Budd 2021; Laycock and Weldon 2019), and Erin O'Toole also explored stoking a divide between “urban cosmopolitans against small‐town values” (Wherry 2020). Poilievre's anti‐elitism, however, elevates such frames to new heights in terms of consistency, frequency, intensity, and hostility to amount to a qualitative difference. Our empirical data have illustrated that in almost every set of criteria identified in the literature on populist “thin” ideology (Mudde and Rovira Kaltwasser 2017) as well as style—anti‐elitism, “bad manners”, performance of crisis, belligerent tone, oversimplification of issues, ordinary language and slogans, and so forth— (Ostiguy et al. 2021), Poilievre fits the bill, and can be said to mark a break in the CPC.

Some of this shift can be explained by a single supply‐side factor: Poilievre is a talented politician and communicator who excels at reaching and mobilizing the public, as agreed on by supporters and critics alike. But there are new “demand‐side” realities as well. The CPC constituency has not remained stable since Harper left power. As Grave and Maher (2022) observe, “older mainstream Tories, motivated by a desire for low taxes and smaller government, have been joined by a younger and angrier set of voters”, who are “far more economically insecure and display higher levels of institutional mistrust than other voters”—according to one poll the authors cite, 66 percent of Conservatives declare “they never trust the government to do the right thing”. Poilievre's CPC speaks directly to this mistrust: A recent X/Twitter‐based content analysis of Canadian MPs’ posts from 2021 to 2025 finds that mistrust‐driven rhetoric not only surged most dramatically among Conservative MPs—who by far outranked all other parties in this regard—, but it also garnered the highest online engagement among Conservative audiences (Vivès et al. 2025).

In the post‐pandemic context of political polarization, economic uncertainty, and cultural anxiety, Poilievre's carefully honed populist framing resonates with sizeable segments of the Canadian public. Data from the 2025 election demonstrate that increasingly, “Canada's new Conservative movement resembles Donald Trump's”, as the Conservative base has shifted geographically and demographically, with growing support among working‐class immigrant voters in suburban ridings (The Economist 2025). Although Poilievre does not substantially deviate from traditional Conservative positions in core ideological terms, he appears to compensate by amplifying an anti‐elite worldview and populist discursive style deliberately crafted to tap into growing public mistrust and to capitalize on emerging constituencies shaped by discontent and volatility.

In terms of organizational strategies, finally, the picture once again becomes more ambiguous, yet a trend is discernible. Poilievre embodies the spirit of Reform's “common sense” populism to forge direct links with the masses through his charismatic leadership, but this has not so far come at the expense of taking over (as Trump has done with the Republican Party) the CPC's traditional party structure, which is neither shapeless nor weak, as the literature might predict (Weyland 2017). At the same time, his “outsider” persona against Ottawa's “Laurentian elites” was used to justify particularly aggressive tone and tactics, arguably more blatant and publicly visible than in the Harper years, against internal rivals and dissenters during the CPC leadership race and its immediate aftermath. Since then, Poilievre has imposed stringent party discipline, with MPs closely monitored by staffers, discouraged from unsanctioned media interactions, and expected to echo the leader's messaging. These tendencies intensified during the 2025 election campaign, when decision‐making was concentrated in a small inner circle, fights were picked with Conservative premiers like Doug Ford and Tim Houston, and the spotlight remained exclusively on Poilievre himself. These are signs that may or may not indicate an evolving shift toward the personalist, centralized control often associated with populist party organization. Party discipline is the norm in Canadian politics rather than the exception, where it is common for MPs across the political spectrum to face pressure to follow the party leadership line (Marland 2020). It thus remains an open question whether these tendencies mark the beginning of a full‐blown populist turn or simply reflect a heightened version of Canada's already centralized party discipline.

Outside his party, the media appears as the most obvious institution that Poilievre energetically undermines and actively bypasses, which overlaps with the preference for “unmediated communication” with the masses attributed to populist strategies (Moffitt 2020, 19). His rare participation in interviews, refusal to attend the Parliamentary Press Gallery Dinner, confrontational tone with reporters, public attacks on media outlets like the CBC, and restrictive campaign practices during the 2025 election all point to an antagonistic posture that favours direct, social media‐based outreach over traditional journalistic channels. In this regard, Poilievre's social media activity as an alternative to journalistic channels, and specifically his prolific production of videos in the various categories we identified, offer a fertile ground for future social scientific analyses that pertain to populism.

As another note on strategies, it should be underlined that our study has focused on the 2022–2025 period, during which Poilievre has become and consolidated his position as the leader of the CPC, Canada's official *opposition* party. How Poilievre and his party would perform if elected to form a government is the subject of another subfield of populism studies, which sheds light on the actions of “populists in power” or the nature of “populist policymaking”. This literature highlights that populist strategies in power create a sense of “permanent mobilization”, continuing to target intermediary institutions that provide checks and balances as working against the general will, downplay technocratic expertise, and prefer polarization over procedure and deliberation in policymaking (Bartha et al. 2020; Pappas 2019b; Urbinati 2019). Whether Prime Minister Poilievre would make his battle against various “gatekeepers” and “wokes” placed in opposition parties, government institutions, and civil society organizations a perpetual strategy of his mode of government is not yet known.

More broadly for the populism scholarship, the study of Pierre Poilievre's CPC leadership underscores the utility of approaching populism as a multidimensional, context‐dependent, and relational phenomenon (Turnbull et al. 2024). By tracing core ideological continuities with Harper‐era conservatism while identifying various shifts in “thin” ideology, style and strategy, our analysis affirms the value of “complex” conceptualizations that allow populist repertoires to emerge unevenly across different domains. Populism, the study demonstrates, is not reducible to ideology, style, or strategy alone, but is best understood through their interplay, which can involve both dissonance and complementarity. Specifically, our analysis supports the literature's emphasis on distinguishing between thin and thick ideologies (Neuner and Wratil 2022), and between ideology and style, without “considering a primacy of one over the other” (Diehl 2024, 28) but treating them as analytically separate yet closely interconnected and often overlapping in practice.

Despite their uneven manifestation, the three types of repertoires can also be mutually reinforcing, Poilievre's case shows. His libertarian‐inflected “thin” populism reframes the CPC's “thicker” conservative program as resistance to the “elites”, while his confrontational rhetoric amplifies this framing and sustains his outsider persona. Strategically, stringent party discipline, media antagonism, and direct digital outreach operationalize this persona, reinforcing both his ideological stance and rhetorical tone. Ideology, therefore, fuels style, style legitimizes strategy, and strategy sustains the force of both, enabling populist appeals to flourish in multifaceted ways. For the “complex” populism scholarship, these dynamics underscore the value of analyzing the relations between the dimensions, as their complementarity can be as politically consequential as their dissonance.

Finally, in contrast to more binary or ideal‐typical models, the Canadian case illustrates how populism can be articulated within established party structures without necessarily overthrowing them, and how rhetorical and strategic populism may intensify even when core policy positions remain relatively stable. These findings call for caution against flattening analyses that equate populism with a wholesale break from traditional party politics or policy orthodoxy, and lend support to perspectives that recognize the “varieties of populism” (Gidron and Bonikowski 2013). The Canadian experience suggests furthermore that right‐wing populism need not rely on overt ethnonationalism or institutional dismantling à la Trump to resonate with mass constituencies (Hunger and Paxton 2022). Instead, it can gain traction through stylistic antagonism, digital bypassing and antagonization of legacy media, and disciplined personalization of leadership—all features Poilievre has deployed to mobilize mistrust and channel discontent. As such, Canada contributes to the comparative literature as a case where populist communication flourishes within a liberal‐democratic setting marked by high party discipline, offering insight into how populism may adapt and evolve in advanced democracies without immediately tipping into illiberalism. Further comparative research is needed to assess whether this represents a durable model of “populism within the mainstream”, or a transitional phase toward more disruptive political transformation.

## Conflicts of Interest

The authors declare no conflicts of interest.

## Endnotes

1 Although other approaches do exist, these three are generally identified as the most widely used ones in the literature today (Diehl 2022).

2 Founded in 1867, the Conservative Party of Canada used the name Progressive Conservative Party of Canada between 1942 and 2003. The term “Progressive” was added at the insistence of John Bracken, Premier of Manitoba and a figure associated with the wider agrarian populist movement of the 1920s, as a condition for accepting the party leadership. In the late nineteenth and early twentieth centuries, the name Liberal‐Conservative Party was also used in some intervals.

3 As Hawkins and Rovira Kaltwasser (2019, 5) write, “for most people … the differences between those who understand populism as a discourse or a thin‐centered ideology are very slight”, which is why the literature often uses “the notions of ‘discourse,’ ‘frame,’ ‘thin‐centered ideology’ and ‘worldview’ somewhat interchangeably” in empirical research.

4 In addition to the historical literature on Canadian conservatism already cited in the previous section, our comparison with the Harper era relies on political and intellectual biographies of the former Prime Minister (Boily 2016; Ibbitson 2015) as well as his own writings (Harper 2018).

5 The “Calgary School” was a group of conservative scholars at the University of Calgary who, from the 1980s to early 2000s, promoted market‐driven policy that highly influenced Reform and Harper's conservatism (Penner 2024).

6 By “bad manners”, Moffitt (2016) refers to intentional violations of political norms through vulgar language, personal insults, or provocative behavior that signal distance from elite decorum and reinforce the leader's authenticity. “Performance of crisis” refers to the sustained dramatization of a sense of emergency or breakdown—economic, cultural, or political—as a way to justify extraordinary responses and maintain ongoing mobilization.

7 While viewing Poilievre's populism as a “discursive strategy more than a strong ideological stance”, Montigny (2025, x, 19) notes that his effort to win over working‐class votes also translates into policy changes (such as CPC's vote in favor of Canada's first federal anti‐scab law in 2024) as well as active campaigning in labour unions and blue‐collar workplaces.

8 #mgtow, or “Men Going Their Own Way”, is a hashtag used by antifeminist men's movements on social networks, common in far‐right groups (Bergeron 2022).
